# Neuroligin-3: A Circuit-Specific Synapse Organizer That Shapes Normal Function and Autism Spectrum Disorder-Associated Dysfunction

**DOI:** 10.3389/fnmol.2021.749164

**Published:** 2021-10-06

**Authors:** Motokazu Uchigashima, Amy Cheung, Kensuke Futai

**Affiliations:** ^1^Department of Cellular Neuropathology, Brain Research Institute, Niigata University, Niigata, Japan; ^2^Department of Neurobiology, Brudnick Neuropsychiatric Research Institute, University of Massachusetts Medical School, Worcester, MA, United States

**Keywords:** autism (ASD), excitatory/inhibitory balance, neuroligin 3 mutation, trans-synaptic adhesion molecule, development, cell adhension molecules, inhibitory synaptic connection, excitatory synaptic activity

## Abstract

Chemical synapses provide a vital foundation for neuron-neuron communication and overall brain function. By tethering closely apposed molecular machinery for presynaptic neurotransmitter release and postsynaptic signal transduction, circuit- and context- specific synaptic properties can drive neuronal computations for animal behavior. Trans-synaptic signaling via synaptic cell adhesion molecules (CAMs) serves as a promising mechanism to generate the molecular diversity of chemical synapses. Neuroligins (Nlgns) were discovered as postsynaptic CAMs that can bind to presynaptic CAMs like Neurexins (Nrxns) at the synaptic cleft. Among the four (Nlgn1-4) or five (Nlgn1-3, Nlgn4X, and Nlgn4Y) isoforms in rodents or humans, respectively, Nlgn3 has a heterogeneous expression and function at particular subsets of chemical synapses and strong association with non-syndromic autism spectrum disorder (ASD). Several lines of evidence have suggested that the unique expression and function of Nlgn3 protein underlie circuit-specific dysfunction characteristic of non-syndromic ASD caused by the disruption of Nlgn3 gene. Furthermore, recent studies have uncovered the molecular mechanism underlying input cell-dependent expression of Nlgn3 protein at hippocampal inhibitory synapses, in which trans-synaptic signaling of specific alternatively spliced isoforms of Nlgn3 and Nrxn plays a critical role. In this review article, we overview the molecular, anatomical, and physiological knowledge about Nlgn3, focusing on the circuit-specific function of mammalian Nlgn3 and its underlying molecular mechanism. This will provide not only new insight into specific Nlgn3-mediated trans-synaptic interactions as molecular codes for synapse specification but also a better understanding of the pathophysiological basis for non-syndromic ASD associated with functional impairment in Nlgn3 gene.

## Introduction

Proper brain function requires the orchestration of 10^15^ synaptic connections that provide platforms for intercellular communication between 10^11^ neurons (Sporns et al., 2005). Chemical synapses which mediate these synaptic connections are characterized as intercellular complex organelles apposing two separated sites containing molecular machinery for presynaptic neurotransmitter release and postsynaptic signal transduction (Biederer et al., 2017). Presynaptic release machinery converts electrical signals propagated as action potentials into chemical signals such as neurotransmitters (Sudhof, 2012). Postsynaptic signal transduction machinery receives neurotransmitters to change postsynaptic excitability via many classes of receptors (Sheng and Kim, 2011). Thus, chemical synapses can provide not only spatiotemporal precision but also diversification of intercellular communication dependent on the pre- and postsynaptic cells and neural activity (O’Rourke et al., 2012). For instance, postsynaptic receptors are basically matched to presynaptic neurotransmitter identity at excitatory and inhibitory synapses (Sheng and Kim, 2011). Hebbian synaptic plasticity, which underlies learning and memory, can regulate the molecular organization of chemical synapses in an input cell-selective manner (Citri and Malenka, 2008). This molecular diversity provides not only distinct synaptic properties at individual synapses but also adequate neuronal computations for animal behaviors. One promising mechanism to generate the molecular diversity of chemical synapses is through trans-synaptic signaling via synaptic cell adhesion molecules (CAMs) at the synaptic cleft (de Wit and Ghosh, 2016).

Neuroligin (*Nlgn*) genes encode single transmembrane postsynaptic CAMs that mediate trans-synaptic signaling for bidirectional synaptic organization. Four Nlgn genes (*Nlgn1*, *Nlgn2*, *Nlgn3*, and *Nlgn4*), and a fifth gene [*Nlgn4X* and *Nlgn4Y (also referred to as Nlgn5*)] in human, have been identified (Ichtchenko et al., 1995, 1996; Bolliger et al., 2001; Bolliger et al., 2008). The extracellular cholinesterase-like domains and intracellular PDZ (postsynaptic density 95/discs large/zona occludens-1)- or gephyrin-binding motif are important for trans and cis protein interactions. Despite structural homology between the four Nlgn proteins, each Nlgn has a distinct pattern of subcellular localization at excitatory, inhibitory, dopaminergic, and cholinergic synapses (Song et al., 1999; Varoqueaux et al., 2004; Takacs et al., 2013; Uchigashima et al., 2016). *Nign1* was the first cloned Nlgn identified as a Neurexin (Nrxn) binding partner (Ichtchenko et al., 1995), and Nlgn1 localizes predominantly at excitatory synapses, and regulates excitatory synaptic transmission and plasticity (Song et al., 1999; Chubykin et al., 2007). Nlgn2 specifically targets inhibitory synapses and controls inhibitory synaptic transmission (Varoqueaux et al., 2004; Poulopoulos et al., 2009). Human and mouse Nlgn4 localize at excitatory and inhibitory synapses, respectively (Hoon et al., 2011; Marro et al., 2019). Therefore, these three Nlgn proteins are exclusively expressed at one type of synapse. In contrast, Nlgn3 is the only Nlgn isoform localized at subsets of both excitatory and inhibitory synapses (Budreck and Scheiffele, 2007; Baudouin et al., 2012; Uchigashima et al., 2020b) and regulates their synaptic functions (Tabuchi et al., 2007; Etherton M. et al., 2011; Shipman et al., 2011; Foldy et al., 2013; Horn and Nicoll, 2018). Nlgn3 is also associated with non-syndromic autism spectrum disorder (ASD), which is characterized by challenges with social communication and restricted behaviors due to unknown etiology. In 2003, the rare R451C substitution in Nlgn3 gene was identified in two brothers diagnosed with ASD (Jamain et al., 2003). Animal models with this missense mutation or other loss-of-function mutations in *Nlgn3* display ASD-associated behavioral phenotypes such as abnormal social interaction, stereotyped behavior, and enhanced spatial learning (Tabuchi et al., 2007; Baudouin et al., 2012; Rothwell et al., 2014). Importantly, conditional gene targeting has identified specific circuits responsible for some ASD-associated behaviors (Baudouin et al., 2012; Rothwell et al., 2014). Therefore, the heterogeneous localization and function of Nlgn3 at distinct synapses is hypothesized to underlie circuit-specific behavioral phenotypes caused by the disruption of *Nlgn3*.

What trans-synaptic signaling is critical for the unique expression and function of Nlgn3? *Nlgn3* generates some alternatively spliced isoforms that can distinctly interact with their binding partners (Sudhof, 2008, 2017b). In particular, Nrxn, which is a major binding partner of Nlgn3, can also make thousands of variants based on the activity of different promoters and multiple alternative splicing events on each Nrxn gene, allowing for interactions between a specific pair of Nlgn3 and Nrxn isoforms (Koehnke et al., 2010). Recently, we found splice isoform- and circuit-dependent functions of Nlgn3 and its underlying molecular mechanism via specific trans-synaptic interactions with presynaptic Nrxns (Uchigashima et al., 2020a, b). These findings suggest that trans-synaptic signaling of specific alternatively spliced isoforms of Nlgn3 and Nrxn isoforms could play an important role in synapse specification at a subset of synapses and contribute to the pathophysiological basis of non-syndromic ASD associated with functional impairment in *Nlgn3*. In this review article, we overview general characteristics of Nlgn3 expression and function and then focus on circuit-specific function of Nlgn3. Comprehensive review articles for Nlgns are available from other groups (Bemben et al., 2015; Jeong et al., 2017; Ali et al., 2020; Nguyen et al., 2020; Kim et al., 2021).

## Molecular Basics of Nlgn3

### Structure of Nlgn3 Gene and Protein

**FIGURE 1:**
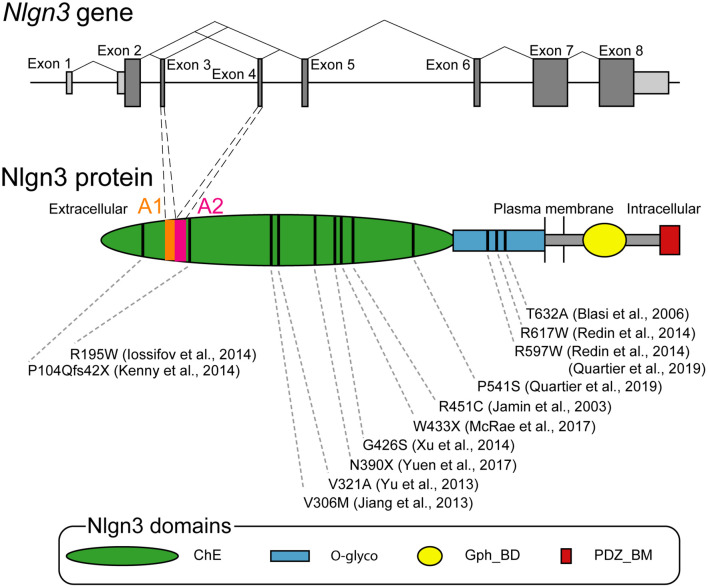
Genomic and protein structures of Neuroligin 3 (Nlgn3). Upper schema showing the organization of human Nlgn3 and splicing patterns at exons 3 and 4. Bars indicate exons with the coding and untranslational regions colored in dark and bright gray, respectively. Lower schema showing the domain structure of Nlgn3. Bars indicate positions of the mutations associated with ASD and schizophrenia. ChE, cholinesterase-like domain, Gph_BD, gephyrin binding domain; O-glyco, O-linked glycosylation sites; PDZ_BM, PDZ domain-binding motif.

*Nlgn3* gene was originally cloned from rat brains as a homolog of rat *Nlgn1* (Ichtchenko et al., 1996). *Nlgn3* is conserved from invertebrates to vertebrates including humans. For instance, human *Nlgn3* shows a 92 or 98% sequence similarity at the transcriptional or translational levels, respectively, compared with the rat ortholog (Philibert et al., 2000). Drosophila *Nlgn* genes (Dnlg1-4) are independently diversified during evolution in invertebrates and vertebrates but have relatively high (approximately 20%) sequence homologies to human *Nlgns* (Knight et al., 2011). Human *Nlgn3* is 32,272 bp in length on an X-chromosome region (Xq13.1) and composed of eight exons ranging from 60 to 1,864 bp (Philibert et al., 2000). The coding region spans from exon 2 to 8. Exons 7 and 8 are the largest exons, encoding about 65% of Nlgn3 protein (Figure 1). *Nlgn3* promotor has a Wnt signal-responsive element, allowing for Wnt-mediated transcriptional control of *Nlgn3* (Medina et al., 2018). Premature transcripts of *Nlgn3* are processed by splicing out particular exons. Exons 3 and 4, encoding 20 amino acid sequences named A1 and A2, can be spliced out, thereby generating four theoretical splice isoforms from *Nlgn3* (Nlgn3-A, +A1, +A2, and +A1A2). Exon 7 may also be alternatively spliced because some *Nlgn3* mRNAs are truncated with a lack of exon 7 (Talebizadeh et al., 2006). The longest transcript of *Nlgn3* is translated into Nlgn3, which is made up of 848 amino acids in humans and rats, partly through postsynaptic local translational machinery (Ichtchenko et al., 1996; Philibert et al., 2000; Cajigas et al., 2012).

Nlgn3 is a single membrane spanning protein with an N-terminal hydrophobic sequence with the characteristics of a cleaved signal peptide followed by a large extracellular domain, a highly conserved single transmembrane region, and a short cytoplasmic domain (Ichtchenko et al., 1996). This molecular structure is common to other Nlgn isoforms, as rat Nlgn1-3 share 52 and > 70% identical residues in the full length and extracellular domain, respectively (Ichtchenko et al., 1996; Fabrichny et al., 2007). The extracellular domain is composed of a large cholinesterase-like domain and O-glycosylated sequence (Figure 1). The cholinesterase-like domain has the α/β-hydrolase fold which is structurally shared with acetylcholinesterase and thyroglobulin (De Jaco et al., 2010). However, the α/β-hydrolase fold found in Nlgns is commonly non-catalytic due to a lack of an active site serine and alternatively responsible for binding to other synaptic molecules. The two alternatively spliced insertions A1 and A2 locate on the extracellular cholinesterase-like domain on Nlgn3. A1 is a sequence homologous to the splice insert A of *Nlgn1* and contains two cysteines, suggesting a possible formation of a disulfide-bonded ring. In contrast, A2 is a sequence homologous to the splice insert A of *Nlgn2* and has no cysteines (Hoffman et al., 2004). Importantly, the presence or absence of the splice insertions A1 and A2 play roles in synaptic function as described previously (Philibert et al., 2000; Oku et al., 2020; Uchigashima et al., 2020b). The extracellular cholinesterase-like domain also has the Nlgn-Nlgn dimerization interface which is highly conserved among different Nlgn isoforms (Dean et al., 2003; Arac et al., 2007; Yoshida et al., 2021).

### Dimerization of Nlgn3 Protein

Nlgn3 molecule forms homodimers and heterodimers with other Nlgn isoforms in the secretory pathway and translocate to the plasma membrane (Poulopoulos et al., 2012; Yoshida et al., 2021). Nlgn1 protein can participate in the heterodimerization with Nlgn3 (Poulopoulos et al., 2012). However, the heterodimer formation of Nlgn2 and Nlgn3 is controversial. Nlgn3 is anatomically co-localized and biochemically co-immunoprecipitated with Nlgn2 (Budreck and Scheiffele, 2007). Nlgn3 functionally requires Nlgn2 at hippocampal inhibitory synapses (Nguyen et al., 2016). These biochemical, anatomical, and functional pieces of evidence support the heterodimer formation of Nlgn2 and Nlgn3. However, an *in situ* chemical cross-linking study failed to detect heterodimers of Nlgn2 and Nlgn3 in hippocampal neurons (Poulopoulos et al., 2012). Dimerization of Nlgns, including Nlgn3, is essential for synapse specification and function (Dean et al., 2003; Ko et al., 2009; Shipman and Nicoll, 2012b). The significance of dimerization on Nlgn trafficking is controversial. Poulopoulos et al. (2012) reported that the lack of dimerization retains Nlgn in the secretory pathway although other groups reported that monomer mutants are able to translocate to the plasma membrane (Ko et al., 2009; Shipman and Nicoll, 2012b).

### Activity-Dependent Cleavage of Nlgn3 Protein

Nlgn3 protein can be cleaved on its juxtamembrane domain in an activity-dependent manner which is conserved from rodents to humans (Bemben et al., 2019). Although Nlgn1 can also be shed during elevated neuronal activity, Nlgn1 does not share the same molecular pathway for cleavage as Nlgn3. The shedding of Nlgn1 depends on NMDA receptor activation, Ca^2+^/calmodulin-dependent protein kinase, and proteolytic activity of a disintegrin and metalloproteinase domain-containing protein 10 (ADAM10) or matrix metalloproteinase 9 (MMP9) (Peixoto et al., 2012; Suzuki et al., 2012). In contrast, ADAM10 claves Nlgn3 under basal condition and activity-dependent cleavage of Nlgn3 is mediated by metabotropic glutamate receptor activation, protein kinase C (PKC) signaling, and MMPs, irrespective of any combination of Nlgn3 dimers (homomers or heteromers) (Kuhn et al., 2016; Venkatesh et al., 2017; Bemben et al., 2019). In addition, Nlgn3 confers sensitivity to PKC-dependent ectodomain shedding of Nlgn1 and Nlgn2 (Bemben et al., 2019). Interestingly, Nlgn3 extracellular ectodomain functions as a cortical neuronal activity-regulated glioma mitogen promoting high-grade glioma (HGG) proliferation and growth (Venkatesh et al., 2015, 2017) (see Section “Nlgn3 in tumors”). However, it remains elusive whether Nlgn3 ectodomain affects the translation of other proteins.

### Association of *Nlgn3* Gene With Autism Spectrum Disorder

*Nlgn3* gene is involved in a non-syndromic monogenic form of ASD. As of today, over ten missense mutations are identified on *Nlgn3* locus in individuals with ASD (Figure 1; Jamain et al., 2003; Blasi et al., 2006; Jiang et al., 2013; Yu et al., 2013; Iossifov et al., 2014; Kenny et al., 2014; Redin et al., 2014; Xu et al., 2014; Deciphering Developmental Disorders, 2017; Yuen et al., 2017; Quartier et al., 2019). Among these mutations, R451C missense mutation has been most intensively studied (Jamain et al., 2003). The estimated occurrence of R451C mutation is < 3% among people with non-syndromic ASD (Quartier et al., 2019). R451C mutation site is located in the helix next to the Nlgn dimerization interface and is important for cell surface trafficking. It does not interfere with dimerization of Nlgn3 mutant protein (Poulopoulos et al., 2012), but shows retention of Nlgn3 mutant in the endoplasmic reticulum (ER), leading to a 90% loss of Nlgn3 from the cell surface (Chih et al., 2004; Comoletti et al., 2004). The mutant protein retained in the ER is preferentially degraded by the proteasome (Trobiani et al., 2018). Additionally, this retention can activate some pathways of the unfolded protein response (UPR) to induce the upregulation of the molecular chaperone BiP (immunoglobulin heavy-chain-binding protein) or ER stress-related transcription factor CHOP [C/EBP (CCAAT/enhancer-binding protein)-homologous protein], supporting a possible molecular mechanism that influences neuronal functions in individuals carrying the R451C mutation of *Nlgn3* gene (Trobiani et al., 2018). Additional missense mutations of the R597W and P514S were reported in a few multiplex families with ASD and intellectual disability (Redin et al., 2014; Quartier et al., 2019). Importantly, all these missense mutations can cause severe loss of Nlgn3 expression at the postsynaptic membrane via abnormal membrane trafficking (Tabuchi et al., 2007; Quartier et al., 2019).

Deletions or frameshift mutations in the coding region of *Nlgn3* were also reported in individuals with ASD (Levy et al., 2011; Sanders et al., 2011; Yuen et al., 2017). Furthermore, single nucleotide variants in the intron or 3′ untranslational region (3′UTR) of *Nlgn3* were identified in a cohort of 144 males with ASD (Steinberg et al., 2012). The intronic variants locate within conserved transcription factor binding sites that could potentially affect gene regulation. The functional impact of the 3′UTR variant remains unclear because no effect on the expression of Nlgn3 was detected by luciferase assay. In addition, *Nlgn3* might be associated with schizophrenia. A rare loss-of-function mutation of *Nlgn3*, which can cause a premature stop 42 codons downstream of the frameshift mutation in exon 2, was also found among a study of 273 people diagnosed with schizophrenia (Kenny et al., 2014).

Dysregulation of mTOR (mammalian target of rapamycin) and MAPK (mitogen-activated protein kinase) pathways are strongly implicated in ASD (Bramham et al., 2016; Winden et al., 2018). eIF4E (eukaryotic translation initiation factor 4E) is an mRNA cap-binding protein that has also been associated with ASD given that eIF4E-mediated translation is the final common process in mTOR, MAPK, and FMRP pathways (Amorim et al., 2018). eIF4E transgenic (TG) mice exhibit abnormal translation, ASD-like behaviors, and synaptic deficits (Santini et al., 2013). Importantly, eIF4E TG or 4E-BP2, an eIF4E repressor, KO mice display abnormal social behaviors and increased *Nlgn* translation (Gkogkas et al., 2013). Furthermore, *Nlgn3* local translation is regulated by fragile X mental retardation protein encoded by fragile mental retardation 1 (*Fmr1*) gene, which is the most common monogenic cause of syndromic ASD (Chmielewska et al., 2019). Taken together, these findings indicate that *Nlgns* are downstream targets of ASD-associated signaling pathways.

### Nlgn3-Mediated Translational Regulation

Recently, Hornberg et al. (2020) reported a novel function of Nlgn3 protein to regulate mRNA translation. Shot-gun proteomics in the ventral tegmental area (VTA) of Nlgn3 KO mice identified altered expression of proteins associated with mRNA translation. Moreover, AHA (methionine analog azidohomoalanine) labeling elucidated the disruption of translation homeostasis in Nlgn3 KO mice. Treatment with ETC-168, an inhibitor of MAP kinase-interacting kinases (MNK) 1/2, ameliorated the dysregulation of mRNA translation and abnormal social behavior (Hornberg et al., 2020). These results clearly indicate that Nlgn3 is not just synaptic glue but acts as a synaptic regulator. Indeed, Nlgn3 regulates dendritic structure by modulating mTOR signaling (Xu et al., 2019) and Nlgn3 KO caused dysregulation of mGluR-dependent signaling (Baudouin et al., 2012; but see Rothwell et al., 2014; Zhang et al., 2015). These studies support the profound importance of Nlgn3 as an upstream regulator of translational pathways vulnerable in ASD.

## Unique Expression of Nlgn3 Protein at Synapses

### Cellular Expression of *Nlgn3* mRNA

*Nlgn1-3* mRNA are highly expressed in the central nervous system (Ichtchenko et al., 1996; Gilbert et al., 2001). The expression levels in rat brains are low at birth and upregulated 2–3 times during postnatal development (Varoqueaux et al., 2006). A modest developmental increase of *Nlgn3* mRNA has been reported based on quantitative RT-PCR of total mRNA levels in the mouse medial nucleus of the trapezoid body (MNTB) (Zhang et al., 2017). *Nlgn3* is expressed in various types of neural cells: neurons, astrocytes, oligodendrocyte precursor cells (OPCs), newly formed oligodendrocytes, and myelinating oligodendrocytes (Zhang et al., 2014; Proctor et al., 2015; Stogsdill et al., 2017). Olfactory ensheathing glia are also known as *Nlgn3*-expressing cells (Gilbert et al., 2001). *Nlgn3* expression levels vary depending on the cell type. In the ventral striatum (nucleus accumbens), there are two different types of projection type neurons called medium spiny neurons (MSNs). *Nlgn3* mRNA levels, but not *Nlgn1* and *Nlgn2*, are significantly higher in D1 dopamine receptor expressing-MSNs than in D2-MSNs (Rothwell et al., 2014). Interestingly, single cell RNAseq demonstrates that *Nlgn3* levels are the highest in OPCs, which make bona fide glutamatergic and GABAergic synapses as postsynaptic cells (Bergles et al., 2000; Lin and Bergles, 2004; Ziskin et al., 2007). *Nlgn3* contains four alternative splice variants (Nlgn3-A, +A1, +A2 and, +A1A2) with different expression levels. Nlgn3-A and Nlgn3+A2 splice isoforms are dominantly expressed in hippocampal CA1 pyramidal cells (Uchigashima et al., 2020b). *Nlgn3* is also detected outside the central nervous system (CNS) (Philibert et al., 2000; Suckow et al., 2008). Rat *Nlgn3* is expressed in pancreatic islet beta cells at lower levels than in the brain (Suckow et al., 2008).

### Subcellular Distribution of Nlgn3 Protein

Consistent with the expression pattern of *Nlgn3* mRNA, Nlgn3 protein is richly expressed in the CNS. In the mouse brain, the expression of Nlgn3 begins as early as E12, peaks during postnatal weeks 2 and 3 when synaptogenesis is maximized, and lasts throughout the adult stage (Budreck and Scheiffele, 2007; Trobiani et al., 2018). Nlgn3 is detectable in both neuronal and non-neuronal cells (Varoqueaux et al., 2006; Proctor et al., 2015; Stogsdill et al., 2017). Some studies further demonstrate the *in vitro* and *in vivo* subcellular distribution of Nlgn3 by immunostaining with specific antibodies in the mouse CNS. Nlgn3 is expressed across the whole brain with variable intensities in distinct regions (Uchigashima et al., 2020b). The expression levels are high in the hippocampus, neocortex, striatum, and brain stem, and low in the thalamus and cerebellum. The hippocampus is the most extensively analyzed area. Immunopositive signals for Nlgn3 are associated with both excitatory and inhibitory synapses in hippocampal primary neurons (Budreck and Scheiffele, 2007) or the adult hippocampal CA1 region (Uchigashima et al., 2020b). Importantly, Nlgn3 is selectively expressed at inhibitory synapses expressing cannabinoid receptor CB1 or vesicular glutamate transporter type 3 (VGT3) [presumably co-expressing cholecystokinin (Cck)], but not at other major inhibitory synapses expressing parvalbumin (Pv) or somatostatin (Sst) in the hippocampal CA1 region (Uchigashima et al., 2020a). Input cell-dependent expression of Nlgn3 is also noted in the cerebellar cortex. Nlgn3 is localized at parallel fiber excitatory synapses, mossy fiber excitatory synapses, and a subset of inhibitory synapses including molecular layer interneuron-Purkinje cell synapses, but not at climbing fiber excitatory synapses (Baudouin et al., 2012; Lai et al., 2021). To date, no reports have addressed the distribution of endogenous Nlgn3 splice isoforms due to technical difficulty. However, Nlgn3 overexpression shows splice isoform-dependent targeting of Nlgn3 protein to distinct synapses as shown in a recent study (Uchigashima et al., 2020b).

### Protein-Protein Interaction of Nlgn3 at Synapses

**FIGURE 2:**
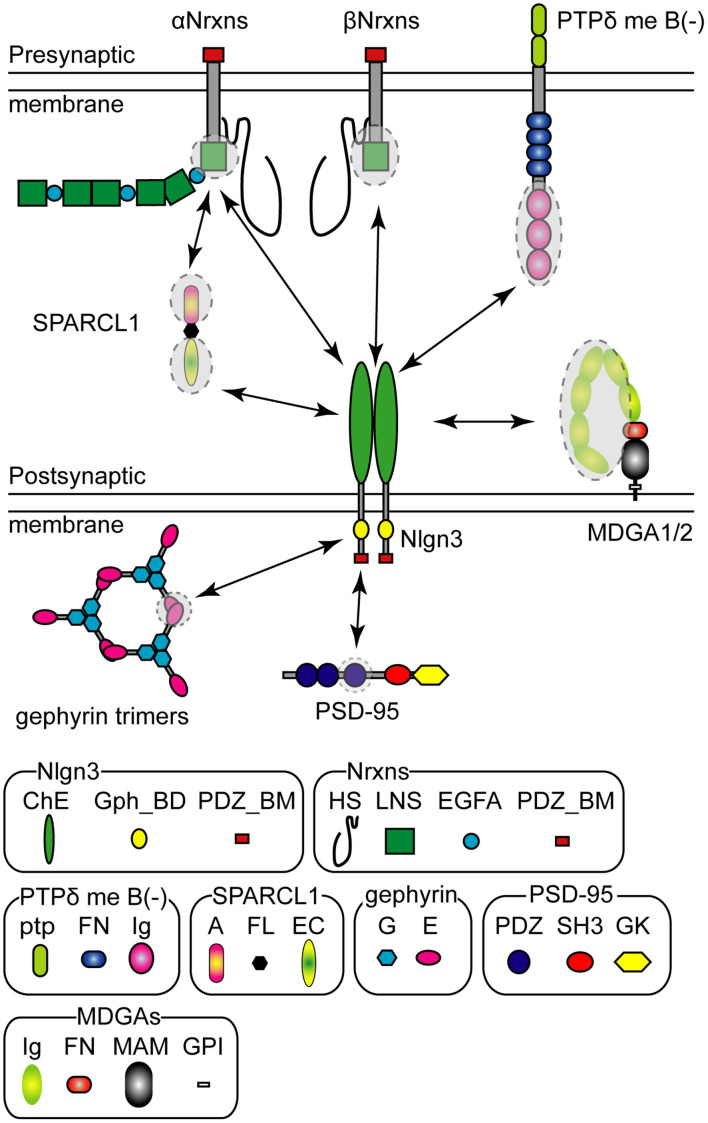
Pre- and postsynaptic Neuroligin 3 (Nlgn3) binding partners. Schematic diagram of the major Nlgn3 binding proteins. Shaded circles with dashed lines indicate the protein domains that interact with Nlgn3. HS, Heparin sulfate; LNS, laminin/neurexin/sex-hormone-binding globulin domain; EGFA, epidermal growth factor-like domains; PDZ_BM, PDZ domain-binding motif; ChE, Cholinesterase-like domain; Gph_BD, Gephyrin binding domain, ptp; protein tyrosine phosphatase domain; FN, fibronectin type III domain; Ig, Ig-like domain; A, acidic domain; FL, follistatin-like domain; EC, EF hand Ca^2+^ binding domain; G, G-domain; E, E-domain, PDZ, PDZ domain; SH3, Src-homology-domain-3; GK, guanylate kinase domain; MA, MAM domain; GPI, glycosylphosphatidylinositol anchor.

The extracellular domain of Nlgn3 protein trans-synaptically binds to that of Nrxns in a Ca^2+^-dependent manner (Ichtchenko et al., 1996). Nrxns are presynaptic CAMs encoded in three genes (*Nrxn1*, *Nrxn2*, and *Nrxn3*) that are transcribed under different promoters as longer alpha (αNrxn1–3), shorter beta (βNrxn1–3), and Nrxn1-specific gamma (γNrxn1) isoforms (Figure 2; Tabuchi and Sudhof, 2002; Sterky et al., 2017). Each Nrxn gene has six alternative splice (AS) sites, named AS1–AS6, potentially generating thousands of Nrxn splice isoforms (Puschel and Betz, 1995; Ullrich et al., 1995; Missler and Sudhof, 1998; Gorecki et al., 1999; Schreiner et al., 2014; Treutlein et al., 2014). Notably, alternative splicing at AS4 is well characterized as a molecular switch that determines trans-synaptic CAM binding pairs. A surface plasmon resonance binding assay has demonstrated the comparative binding affinity of Nlgn proteins to βNrxn protein. Nlgn3 weakly binds to βNrxn compared with Nlgn1 and Nlgn2 with ∼10-fold lesser binding strength irrespective of the splice isoform of either binding partner (Koehnke et al., 2010). All Nlgn3 splice isoforms exhibit a higher binding affinity to βNrxn without the insertion of AS4 than those with AS4 (Ichtchenko et al., 1996; Koehnke et al., 2010). Structural analysis predicts the βNrxn1 binding site of Nlgn3 protein is composed of H279-E281, N362, and Q370-N377 (Yoshida et al., 2021). The ASD-associated R451C mutation on the extracellular domain can interfere with this binding (Comoletti et al., 2004; Yoshida et al., 2021). Nlgn3 can also bind to αNrxn1 protein regardless of the presence or absence of the splice insertion at AS4 (Boucard et al., 2005). Interestingly, a specific trans-synaptic interaction between Nlgn3-A and αNrxn1+AS4 controls inhibitory synaptic transmission in an input cell-dependent manner, as described in detail below (see section “Molecular Code” for an Input Cell-Dependent Function of Nlgn3 Protein at Hippocampal Inhibitory Synapses) (Uchigashima et al., 2020a). To the best of our knowledge, there are no comparative studies examining the binding affinity of Nlgn3 to αNrxn. Recently, heparin sulfate (HS) modification of Nrxn was reported to mediate trans-synaptic interactions with Nlgn1–4 proteins and regulate their synaptic functions (Zhang et al., 2018). The three lysine and arginine residues of human Nlgn3 (K609, R611, and R613), which are conserved across different Nlgn isoforms and animal species, are required for HS binding for full Nrxn binding, but not for the dimerization or membrane trafficking of Nlgn3 (Zhang et al., 2018).

Nrxn has been considered the sole presynaptic binding partner of Nlgn for decades, however, a recent study identified Nlgn3 as a selective ligand for presynaptically-expressed type IIA receptor protein tyrosine phosphatase delta (PTPδ) (Yoshida et al., 2021). Similar to Nrxn genes, PTPδ has multiple isoforms generated by alternative splicing of microexons (Takahashi and Craig, 2013). Yoshida et al. (2021) demonstrated that PTPδ lacking mini-exon B can trans-synaptically interact with Nlgn3 and influence presynaptic differentiation (Figure 2). Interestingly, a structural analysis predicted a direct interaction of G371, E372, L374, and N375 in the extracellular domain of Nlgn3 with the third immunoglobulin domain of PTPδ protein, suggesting that PTPδ and βNrxn1 potentially compete for binding to Nlgn3. Importantly, the Nlgn3 binding interface to PTPδ is not present in other Nlgns, further highlighting the versatile function of Nlgn3 compared with other Nlgns. This non-canonical Nlgn3-PTPδ signaling is hampered by the R451C mutation of Nlgn3 (Yoshida et al., 2021).

MAM domain-containing glycosylphosphatidylinositol anchor 1 (MDGA1) and 2 (MDGA2) proteins also compete for the Nrxn binding site on the extracellular domain of Nlgn3 via a *cis* interaction, blocking trans-synaptic interactions between Nlgn and Nrxn (Lee et al., 2013; Pettem et al., 2013; Connor et al., 2016; Elegheert et al., 2017; Gangwar et al., 2017; Figure 2). The binding affinity of each MDGA protein varies depending on the Nlgn isoform. Both MDGAs exhibit low binding affinity to Nlgn3 compared with Nlgn1 and Nlgn2 (Lee et al., 2013; Elegheert et al., 2017). Furthermore, the heterodimer of Nlgn3 with other Nlgn isoforms likely blocks MDGA-mediated blockage of Nrxn-Nlgn interactions because the immunoglobulin-tandem structure of MDGA is not able to bind to Nlgn heterodimers efficiently (Gangwar et al., 2017). Therefore, MDGA protein-mediated modulation would be less effective at Nlgn3-containing synapses, compared with Nlgn3-lacking synapses.

Secreted protein acidic and rich in cysteine-like 1 (SPARCL1 also known as Hevin) is an extracellular matrix released from astrocytes and important for excitatory synaptogenesis (Kucukdereli et al., 2011). Interestingly, SPARCL1 forms synaptic triads with αNrxn1 and Nlgn1, and Nlgn3 can be incorporated into this complex (Kucukdereli et al., 2011; Singh et al., 2016; Figure 2). However, the molecular mechanism underlying the synaptogenic effect of SPARCL1 remains controversial. Gan and Sudhof (2020) reported that SPARCL1 regulates excitatory synaptogenesis independent of Nlgns and Nrxns. Further studies are required to elucidate the physiological role of Nrxn-SPARCL1-Nlgn complexes on excitatory synapses.

The short intracellular domain of Nlgn3 contains the PDZ- (Irie et al., 1997) and gephyrin-binding motifs (Poulopoulos et al., 2009; Figure 2). Both PDZ- and gephyrin-binding motifs are conserved across all Nlgn isoforms, suggesting that all Nlgn isoforms can be potentially delivered into both excitatory and inhibitory synapses. Nlgn3 has some conserved phosphorylation sites in the intracellular domain. In the case of Nlgn1 and Nlgn2, the phosphorylation of the conserved tyrosine residue is functionally critical for excitatory and inhibitory synapses, respectively (Poulopoulos et al., 2009; Giannone et al., 2013). However, the functional roles of Nlgn3 phosphorylation sites remain unclear because no effects of the corresponding Nlgn3 phosphomutants have been detected on synaptic function.

## Normal Function and Autism Spectrum Disorder-Associated Dysfunction of Nlgn3 Protein at Synapses

Nlgn3 protein has been characterized as a synaptic organizer, akin to other Nlgn isoforms. Nlgn3 can induce presynaptic differentiation in co-culture assay with primary neurons and heterologous cells (Scheiffele et al., 2000; Chubykin et al., 2005). Artificial postsynaptic clustering of Nlgn3 can also co-aggregate excitatory postsynaptic proteins including PSD95 or AMPARs but not inhibitory postsynaptic ones (Graf et al., 2004). Thus, these experiments to induce artificial synapses propose bidirectional synaptogenic activity of Nlgn3. However, a functional analysis using physiological synapses is required for a better understanding of the synaptic functions of Nlgn3. Genetic manipulation of *Nlgn3* gene is a powerful tool to reveal the functional role of Nlgn3 at intact synapses. Past functional analysis of Nlgn3 has been performed based on overexpression, knockdown (KD), and KO of Nlgn3 gene/protein or knock-in (KI) of *Nlgn3* mutants, revealing that Nlgn3 can regulate synaptic function in an age-, region-, cell type- and animal model-dependent manner.

### Synaptic Transmission and Structure

One of the most important outcomes obtained by Nlgn3 mutant studies is the heterogeneity of mutation impact in the brain circuits. Three mutant mouse models, including knockout and missense ASD mutations, have revealed that different Nlgn3 mutations cause distinct abnormalities at synapses. This section highlights the differential impact of Nlgn3 mutations in excitatory and inhibitory synapses (Tables 1, 2).

**TABLE 1:** Summary of electrophysiological and other phenotypes in Nlgn3 mutant mouse lines in the hippocampus and cerebellum.

Mouse (source)	Region	Electrophysiological phenotype	Other phenotypes	References
KO (Sud^2^)	Hi Py	mEPSC freq ↓ /amp ↔, NMDA/APMA ↔, PPR ↔		Etherton M. et al., 2011
		mIPSC freq ↑ /amp ↔		
Global cKO (Sud^3^)	Hi Py*	mEPSC freq ↔ /amp ↔		Chanda et al., 2017
		mIPSC freq ↔ /amp ↔		
P0 cKO (Sud^3^)	Hi Py	NMDA/AMPA ↔		Jiang et al., 2017
		mIPSC freq ↑ /amp ↔		
P21 cKO (Sud^3^)	Hi Py	NMDA/AMPA ↔, PPR ↔		
		mIPSC freq ↔ /amp ↔		
R451C KI (Sud^1^)	Hi Py	mEPSC freq ↑ /amp ↔, NMDA/AMPA ↑, PPR ↔,	GluN2B ↑, PSD95 ↑, dendritic branching ↑	Etherton M. et al., 2011
		LTP ↑, NMDA-EPSC amp ↑ /decay ↑	spine size ↓, terminal size ↓	
		mIPSC freq ↔ /amp ↔		
R451C KI (Hei)	Hi Py	NMDA/AMPA ↑		
R451C KI (Sud^1^)	Hi CA3 Py	mEPSC freq ↔ /amp ↔		Pizzarelli and Cherubini, 2013
		mIPSC freq ↑ /amp ↔		
R704C KI (Sud^4^)	Hi Py	mEPSC freq ↓ /amp ↔, NMDA/AMPA ↑, PPR ↔, LTP ↔	VGluT1 ↔, GluA1 ↑, GluA3 ↑	Etherton M. R. et al., 2011
		mIPSC freq ↔ /amp ↔	VIAAT ↔	
KO (Sud^2^)	Hi Py	Pv-IPSC amp ↓	Normal morphology in Pv+ In	Foldy et al., 2013
	Hi Py	Cck-IPSC amp ↑	No morphology in Cck+ In	
		Impaired tonic eCB signaling		
Pv+ cKO (Sud^3^)	Hi Pv+	mEPSC freq ↔ /amp ↔, NMDA/AMPA ↓, PPR ↓	PSD95 ↔	Polepalli et al., 2017
		sEPSC freq ↑ /amp ↔, eEPSC amp ↑		
		Impaired Group III mGluR activity		
		mIPSC freq ↔ /amp ↔		
KO (Tan)	Cb PC	mEPSC freq ↔ /amp ↓	PF synapse morphology →, ectopic CF synapse ↑, (Baudouin et al., 2012)	
		PF-EPSC mGluR-LTD ↓, PPR ↔	GluA2 phosphorylation ↓, mGluR1a ↓	
PC cKO (Sud^3^)	Cb PC	CF-EPSC amp ↓ /PPR ↔		Zhang et al., 2015
		PF-EPSC PPR ↔		
R451C KI (Sud^1^)	Cb PC	mEPSC freq ↑ /amp ↔	(Trobiani et al., 2018)	
R451C KI (Sud^1^)	Cb PC	mEPSC freq ↔ /amp ↔	PC number ↔	Lai et al., 2021
		Impaired synaptic elimination	Normal morphology of PC dendrites	
		mIPSC freq ↔ /amp ↑	Molecular layer In number ↔	
PC cKO (Sud^3^)	Cb PC	mIPSC freq ↔ /amp ↔		Zhang et al., 2015

*Cb:,cerebellum; CF, climbing fiber; eCB, endocannabinoid; Hi, hippocampus; In, interneuron; PC, Purkinje cell; PF, parallel fiber; PPR, paired pulse ratio; Pv+, parvalbumin+ interneuron; Py, pyramidal neuron (^∗^ indicates primary cultures); freq, frequency; ↑, increased; ↓, decreased; ↔, not significant.*

*Sud^1^: (Tabuchi et al., 2007), Sud^2^: (Tabuchi et al., 2007), Sud^3^: (Rothwell et al., 2014), Sud^4^ (Etherton M. R. et al., 2011), Hei: (Chadman et al., 2008), Tan: (Tanaka et al., 2010).*

**TABLE 2:** Summary of electrophysiological and other phenotypes in Nlgn3 mutant mouse lines in other brain regions.

Mouse (source)	Region	Electrophysiological phenotype	Other phenotypes	References
R451C KI (Sud^1^)	SCx Py	mEPSC freq ↔ /amp ↔	VGT1 ↔, synapse number ↔	Tabuchi et al., 2007
R451C KI (Sud^1^)		mEPSC freq ↓ /amp ↔, NMDA/APMA ↔		Etherton M. et al., 2011
R451C KI (Sud^1^)	SCx Py	Pv-IPSC amp ↓ /PPR ↑		Cellot and Cherubini, 2014
KO (Sud^2^)	SCx Py	mIPSC freq ↔ /amp ↔	VIAAT ↑	Tabuchi et al., 2007
R451C KI (Sud^1^)	SCx Py	mIPSC freq ↔ /amp ↑	VIAAT ↑, synapse number ↔	Etherton M. et al., 2011
		mIPSC freq ↑ /amp ↔		
R451C KI (Sud^1^)	SCx Py	mIPSC freq ↑ /amp ↔		Speed et al., 2015
		Impaired tonic eCB signaling		
		Sst-IPSC amp ↔	Sst+ In number ↔	
		Pv-IPSC amp ↔	Pv+ In number ↔	
R451C KI (Sud^1^)	SCx Pv+	eEPSC amp ↔		Cellot and Cherubini, 2014
R451C KI (Sud^1^)	SCx Sst+	Py-EPSC amp ↔		Speed et al., 2015
	SCx Pv+	Py-EPSC amp ↔		
R451C KI (Sud^1^)	BA Py	mEPSC freq ↔ /amp ↑		Hosie et al., 2018
		mIPSC freq ↔ /amp ↓		
KO (Sud^2^)	St D1	mEPSC freq ↔ /amp ↔		Rothwell et al., 2014
	St D1	mIPSC freq ↓ /amp ↔		
	St D2	mEPSC freq ↔ /amp ↔		
	St D2	mIPSC freq ↔ /amp ↔		
KO (Sud^2^)	MNTB	Calyx-EPSC amp ↔, PPR ↔, RT ↔, DT ↔		Zhang et al., 2017
R451C KI (Sud^1^)		Calyx-EPSC amp ↓, PPR ↔, RT ↔, DT ↔		
R704C KI (Sud^4^)		Calyx-EPSC amp ↑, PPR ↔, RT ↔, DT ↔		
Krox20 cKO (Sud^3^)		Calyx-EPSC amp ↓, RT ↔, DT ↔		
Pv cKO (Sud^3^)		Calyx-EPSC amp ↓, RT ↑, DT ↑		
KO (Tan)	VTA DA	GluA2-lacking AMPA-transmission ↑		Bariselli et al., 2018
DAN-KD		GluA2-lacking AMPA-transmission ↑		

*amp, amplitude; BA, basal amygdala; D1, D1R+ medium spiny neuron; D2, D2R+ medium spiny neuron; DA, dopaminergic neuron; eCB, endocannabinoid; In, interneuron; MNTB, medial nucleus of the trapezoid body; Pv+, parvalbumin+ interneurons; Py, pyramidal neurons; SCx, somatosensory cortex; Sst+, somatostatin+ interneurons; St, striatum; VTA, ventral tegmental area; DT, decay time; freq, frequency; PPR, paired pulse ratio; RT, rise time; ↑, increased; ↓, decreased; ↔, not significant.*

*Sud^1^: (Tabuchi et al., 2007), Sud^2^: (Tabuchi et al., 2007), Sud^3^: (Rothwell et al., 2014), Sud^4^ (Etherton M. R. et al., 2011), Tan: (Tanaka et al., 2010).*

#### Excitatory Synapses

A major function of Nlgn3 protein at synapses is to control AMPAR-mediated basal excitatory transmission. Excitatory synapses on hippocampal CA1 pyramidal neurons have been the best characterized so far. Overexpression of Nlgn3 enhances AMPAR-mediated excitatory transmission and expression of presynaptic vesicular glutamate transporter 1 regardless of the specific Nlgn3 splice isoform (Uchigashima et al., 2020b). In Nlgn1/2/3 triple KO neurons, overexpression of Nlgn3+A2 selectively rescues deficits in AMPAR-mediated excitatory postsynaptic current (AMPAR-EPSC) amplitudes, but not NMDAR-EPSC amplitudes, suggesting a functional role of Nlgn3 in AMPAR-mediated excitatory transmission (Chanda et al., 2017). However, global KO of *Nlgn3* gene results in no change in excitatory synaptic transmission (Foldy et al., 2013) or a small decrease in mEPSC frequency but not amplitude (Etherton M. et al., 2011). Conditional deletion of *Nlgn3* starting at P0 or P21 has no effects on excitatory synaptic responses (Jiang et al., 2017). These mild or less significant phenotypes may be caused by a functional redundancy of Nlgn1 and Nlgn3. Indeed, a significant reduction in AMPAR-mEPSC amplitudes is observed in Nlgn1/3 double KO neurons, but not in Nlgn1 KO or Nlgn3 KO neurons (Chanda et al., 2017). In contrast to the moderate phenotype observed in Nlgn3 KO mice, a clear gain-of-function or loss-of-function phenotype is noted for AMPAR-mediated excitatory synaptic transmission in CA1 pyramidal cells of Nlgn3-R451C or -R704C KI mice. Nlgn3-R704C KI is a model of non-syndromic ASD mimicking the mutation identified in *Nlgn4* (Yan et al., 2005). Nlgn3-R451C and -R704C mutations cause 90 and 30% loss of Nlgn3 expression, respectively. Nlgn3-R451C KI mice exhibit a large increase in AMPAR-mediated excitatory synaptic transmission in the hippocampal CA1 region without any changes in presynaptic release probability and the expression of excitatory synaptic proteins (Etherton M. et al., 2011; Hosie et al., 2018). Conversely, the R704C mutation causes a major and selective decrease in AMPAR-mediated synaptic transmission, without any change in NMDAR- or GABA_*A*_R-mediated synaptic transmission, and without detectably altering presynaptic neurotransmitter release (Etherton M. R. et al., 2011).

Interestingly, the impact of Nlgn3 mutations in the calyx-MNTB synapses are distinct from hippocampal synapses. The calyx synapse is a large excitatory synapse in the MNTB that functions in the auditory system. Nlgn3 KO has no effect on excitatory synaptic transmission at the calyx synapse. In contrast, conditional deletion of *Nlgn3* at the late developmental phase causes a large decrease in postsynaptic AMPARs at the calyx synapse without any change in AMPAR composition (Zhang et al., 2017). Nlgn3-R451C and -R704C KI mutants display decreased and increased excitatory synaptic transmission, respectively (Zhang et al., 2017). In the cerebellum of Nlgn3 KO mice, climbing fiber synapses on Purkinje cells exhibit a decrease in AMPAR-EPSC amplitudes (Zhang et al., 2015). In the basolateral amygdala, an increase of excitatory transmission is observed in Nlgn3-R451C KI mice.

Nlgn3 protein contributes to NMDAR-mediated basal synaptic transmission at excitatory synapses on hippocampal CA1 inhibitory interneurons (Polepalli et al., 2017). The Nlgn3 deletion in CA1 Pv+ inhibitory interneurons selectively causes a reduction in postsynaptic NMDAR-mediated synaptic transmission, but not in postsynaptic AMPAR-mediated synaptic currents. Different from CA1 Pv+ interneurons, normal Nlgn3 unlikely contributes to NMDAR-mediated synaptic transmission in CA1 pyramidal cells (Etherton M. et al., 2011). In contrast, the R451C mutation can enhance NMDAR-mediated synaptic transmission, which may be partly caused by enhanced expression of GluN2B-containing receptors (Etherton M. et al., 2011). Since both CA1 pyramidal neurons and Pv+ interneurons receive the same excitatory inputs from CA3 pyramidal neurons, these results indicate that Pv+ interneurons and pyramidal neurons have distinct postsynaptic molecular architectures that contribute to different roles of Nlgn3 on NMDAR function. Nlgn3-R451C and -R704C missense mutations exhibit no major changes in hippocampal or cortical synapse size and density (Tabuchi et al., 2007; Etherton M. et al., 2011; Etherton M. R. et al., 2011). These findings suggest that Nlgns do not have a primary function in synapse formation as reported previously (Varoqueaux et al., 2006). Since Nlgns share their binding partners with other synaptic CAMs, this redundancy may mask the phenotype in loss-of-function of Nlgn3.

These results indicate that ASD-associated Nlgn3 mutations cause circuit-dependent abnormal excitatory synaptic efficacy. The molecular mechanism underlying the differential roles of Nlgn3 on AMPARs and NMDARs is not elucidated. Post-translational modifications of Nlgn3 may contribute to synaptic specification, leading to distinct effects of Nlgn3 on AMPAR- and NMDAR-mediated excitatory transmission. Further analysis is required to highlight the functional impact of Nlgn3 on receptor-mediated synaptic function.

#### Inhibitory Synapses

Similar to studies examining excitatory synaptic function, there are a number of studies that have elucidated the global roles of Nlgn3 protein on GABA_*A*_R-mediated inhibitory synapses. In CA1 pyramidal neurons, global KO or P0 conditional KO of *Nlgn3* gene causes an increase in mIPSC frequency but not amplitude (Etherton M. et al., 2011; Jiang et al., 2017). This phenotype can be interpreted as an increase in presynaptic release probability at GABAergic synapses. Overexpression approach in CA1 pyramidal neurons elucidated that inhibitory synaptic transmission is potentiated by Nlgn3-A and Nlgn3+A2 overexpression and suppressed by Nlgn3+A1 and Nlgn3+A1A2 in the hippocampal CA1 region (Uchigashima et al., 2020b).

Interneurons exhibit extraordinary morphological, physiological and molecular diversity (Markram et al., 2004; Somogyi and Klausberger, 2005; Klausberger and Somogyi, 2008; Pelkey et al., 2017; Booker and Vida, 2018). These classes are distinguished by their distinct peptide expression (Cck+, Pv+, and Sst+), morphologies, and synaptic impact. Recent emerging studies have revealed that Nlgn3 regulates GABA_*A*_R-mediated basal synaptic transmission at specific inhibitory synapses.

Input cell-specific stimulation in Nlgn3 KO mice shows a significant increase in synaptic strength at Cck+ synapses, but no alteration at Pv+ synapses, suggesting an input cell-dependent function of Nlgn3 at hippocampal inhibitory synapses. Interestingly, this selective strengthening of Cck+ synapses is caused by disruption of tonic endocannabinoid signaling via cannabinoid CB1 receptors which are expressed at Cck+ synapses (Foldy et al., 2013). Importantly, this disruption of tonic endocannabinoid signaling also enhances synaptic transmission at Cck+ inhibitory synapses in Nlgn3-R451C KI mice (Foldy et al., 2013), suggesting that Nlgn3 KO and R451C KI mice share common phenotypes at Cck+ inhibitory synapses in the hippocampal CA1 region. In contrast, no change in either mIPSC amplitude or frequency was found in Nlgn3 conditional KO mice at P21 (Jiang et al., 2017), suggesting that Nlgn3 at the early developmental stage is essential for tonic endocannabinoid signaling. As different synaptic phenotypes have been previously reported in global and sparse reductions of single *Nlgn1* or *Nlgn2* expression (Kwon et al., 2012; Uchigashima et al., 2016), sparse KD of *Nlgn3* at an early developmental stage (1–2 days after the preparation of rat or mouse hippocampal slice cultures) was found to suppress inhibitory synaptic transmission at VGT3+ synapses (a subpopulation of CB1+/Cck+ synapses) but not Pv+ synapses (see section “Molecular Code” for an Input Cell-Dependent Function of Nlgn3 Protein at Hippocampal Inhibitory Synapses) (Uchigashima et al., 2020a). Considering a cell-autonomous function of *Nlgn*s (Chanda et al., 2017), these inconsistent results from global and sparse deletions of *Nlgn3* may be partly explained by abnormal network activity in global deletions of *Nlgns*.

Nlgn3 loss-of-function analyses also affect spontaneous miniature inhibitory synaptic events in other brain regions. In the somatosensory cortex, inhibitory synaptic transmission is significantly increased in Nlgn3-R451C KI mice but not in Nlgn3 KO mice (Tabuchi et al., 2007; Speed et al., 2015). This increase is consistent with elevated expression of gephyrin and vesicular inhibitory amino acid transporter (VIAAT, also known as vesicular GABA transporter) and is partly caused by impaired endocannabinoid signaling at CB1-expressing Cck+ synapses (Tabuchi et al., 2007; Speed et al., 2015). In contrast, no change or a reduction in inhibitory transmission is found at either Sst+ synapses or Pv+ synapses on pyramidal cells (Cellot and Cherubini, 2014; Speed et al., 2015). On the other hand, Nlgn3-R451C KI mice exhibit a decrease in mIPSC amplitude in the basolateral amygdala (Hosie et al., 2018).

In the nucleus accumbens, Nlgn3 KO selectively reduces mIPSC frequency by 50% in D1-MSNs but not in D2-MSNs, consistent with cell type-specific expression of *Nlgn3* in D1-MSNs (Rothwell et al., 2014). Furthermore, this reduction of mIPSC frequency on D1-MSNs is commonly observed in R451C KI mice, suggesting a common dysfunction of striatal inhibitory circuits in both Nlgn3 KO and Nlgn3-R451C mice (Rothwell et al., 2014).

In cerebellar Purkinje cells, single KO of *Nlgn2* or *Nlgn3* has no or a weak phenotype in inhibitory synaptic transmission, whereas Nlgn2/3 double KO causes dramatic decreases in both mIPSC frequency and amplitude, suggesting functional redundancy between Nlgn2 and Nlgn3 (Zhang et al., 2015). In contrast, Nlgn3-R451C KI mice increase mEPSC frequency in Purkinje cells. This abnormal increase can be sufficiently rescued by inhibition of a branch for the UPR (Trobiani et al., 2018).

### Synaptic Plasticity and Circuit Remodeling

A few studies have examined the impact of Nlgn3 protein on long-term synaptic plasticity. Parallel fiber-Purkinje cell synapses in the cerebellum can induce mGluR-mediated long-term depression (LTD) which underlies motor coordination. The effect of Nlgn3 KO on this form of LTD remains controversial. Baudouin et al. (2012) reported that constitutive Nlgn3 KO mice exhibited a loss of mGluR-mediated LTD. However, Zhang et al. (2015) showed no change in mGluR-mediated LTD in pan Nlgn-deficient Purkinje cells. Nlgn3 KD caused no significant change on Nlgn1-mediated long-term synaptic potentiation (LTP) at excitatory synapses in the hippocampal CA1 region (Shipman and Nicoll, 2012a).

In contrast to Nlgn3 KO or KD, Nlgn3-R451C mutation significantly affects long-term synaptic plasticity with variable phenotypes at distinct synapses. LTP is enhanced at hippocampal excitatory synapses (Etherton M. et al., 2011). On the other hand, LTD is impaired at corticostriatal excitatory synapses (Martella et al., 2018). Interestingly, the impairment in this form of LTD can be partly rescued by the activation of cannabinoid CB1 receptor, suggesting a dysfunction in endocannabinoid signaling in Nlgn3-R451C KO mice as demonstrated at hippocampal inhibitory synapses (Foldy et al., 2013; Martella et al., 2018).

A functional impact of Nlgn3 in remodeling of neuronal circuits has been reported. Nlgn3 can affect the turnover of spines that form synaptic structures with excitatory neurons. In layer 2/3 pyramidal neurons of the somatosensory cortex, Nlgn3-R451C KI mice show enhanced dynamics of PSD95+ spines, which may be subsequently associated with stable synaptic connectivity. Importantly, abnormal PSD95+ spine dynamics is shared with another mouse model of non-syndromic ASD in which the chromosomal region corresponding to human 15q11–13 is paternally duplicated (patDp/ + mice) (Isshiki et al., 2014).

Nlgn3 also contributes to the developmental elimination of climbing fiber-Purkinje cell synapses. Nlgn3-R451C KI mice impair elimination of redundant climbing fiber-Purkinje cell synapses from postnatal day 10–15 (Lai et al., 2021). Nlgn3 KO mice exhibit ectopic climbing fiber synapses through hetero-synaptic competition with parallel fiber synapses onto Purkinje cells (Baudouin et al., 2012). Therefore, the dysfunction of Nlgn3 likely causes abnormal remodeling of neuronal circuits which can be a major endophenotype of ASD.

### Behavioral Phenotypes of Nlgn3 Mutations in Non-syndromic Autism Spectrum Disorder Animal Models

One prevailing mechanism describing the pathophysiology of ASD is the imbalance between excitation and inhibition in neurons, which can be caused by circuit dysfunction described above. Supporting this notion, emerging evidence has revealed that functional impairment in Nlgn3 gene generates dysfunction in specific circuits. Based on the DSM-5, the diagnostic criteria to characterize ASD are (1) persistent deficits in social communication and interaction and (2) restricted, repetitive patterns of behavior, interests, or activities (American Psychiatric Association, 2013). Many behavioral studies investigating Nlgn3 mutant mouse and Drosophila (Yost et al., 2020) models corroborate the relevance of Nlgn3 dysfunction with ASD-like behavioral features and reveal roles for Nlgn3 in specific brain regions responsible for these behaviors (Table 3). The first study on Nlgn3-R451C, which mimics the human single nucleotide polymorphism (SNP) identified in individuals with ASD, opened the field for investigation of Nlgn3 mutants in ASD-associated phenotypes (Jamain et al., 2003; Tabuchi et al., 2007). This KI line displayed impaired sociability and learning and memory, which was further confirmed in follow-up studies (Etherton M. et al., 2011; Jaramillo et al., 2014). In addition, Nlgn3-R451C KI mice display increased repetitive behavior and aggressiveness, deficits in transitive inference, and abnormal wake and sleep EEG power spectral profiles (Burrows et al., 2015; Liu et al., 2017; Hosie et al., 2018; Norris et al., 2019). Nlgn3 KO mice also display behavioral abnormalities including hyperactivity, normal sociability but deficits in social recognition, reduced ultrasound vocalization, impaired learning and memory, and abnormal pheromone preference (Radyushkin et al., 2009; Dere et al., 2018). Of note, there are inconsistencies in behavioral phenotypes observed among Nlgn3 mutant lines. For example, different Nlgn3-R451C KI lines have distinct sociability and learning and memory phenotypes (Tabuchi et al., 2007; Chadman et al., 2008). Even Nlgn3 KO mouse lines displayed different behavioral phenotypes. These conflicts have been considered due to different genetic backgrounds (Jaramillo et al., 2014, 2018). However, other factors, like housing environments, may also contribute to the inconsistent results (Burrows et al., 2017, 2020; Kalbassi et al., 2017). For example, social environment can affect social submissive behaviors or hierarchy. Male Nlgn3 KO mice under mixed genotype housing with male wild type littermates are submissive to unfamiliar wild type mice, whereas those under single genotype housing are not (Kalbassi et al., 2017). This indicates that the patterns of animal housing could potentially change behavioral results.

**TABLE 3:** Summary of behavioral tests in Nlgn3 mutant mouse lines.

Mouse (source)	Social	Cognitive	Rep.	Motor	Other phenotypes	References
KO (Bro)	Sociability: ↔	FL :↓		Activity: ↑	USV: ↓	Radyushkin et al., 2009
	Social rew/mem: ↓ MWM: ↔				Olfactory-dependent behavior: ↓	
KO (Bro)	NT	NT	NT	NT	Pheromone preference: ↓	Dere et al., 2018
KO (Bro)	Abnormal visual transitive inference.					Norris et al., 2019
KO (Sud^2^)	NT	NT	↑	Activity: ↑		Rothwell et al., 2014
KO (Tan)	Sociability: ↓	NT	NT	NT	Gamma osc: ↓	Modi et al., 2019
	Social rew/mem: ↓					
KO (Tan)	NT	NT	NT	Motor coordination: ↓		Baudouin et al., 2012
				(rescued by Nlgn3 OE in PC)		
KO (Tan)	Housing environment causes different behavioral phenotypes.					Kalbassi et al., 2017
KO (Tan)	Sociability: ↓	NT	NT	NT		Bariselli et al., 2018
	Social rew/mem: ↓					
KD in DA	Sociability: ↓	NT	NT	NT		
	Social rew/mem: ↓					
KO (Tan)	Sociability: ↔	NT	NT	NT		Hornberg et al., 2020
	Social rew/mem: ↓					
KD in DA	Sociability: ↔	NT	NT	NT		
	Social rew/mem: ↓					
	Rescued by MNK inhibition					
cKO (Sud^3^, D1)	NT	NT	↑	Activity: ↑		Rothwell et al., 2014
cKO (Sud^3^, D2)	NT	NT	↔	Activity: ↔		
cKO (Sud^3^, PC)	NT	NT	↔	Activity: ↑		
cKO (Sud^3^, Pv+)	NT	Abnormal fear extinction	Gamma osc: ↓			Polepalli et al., 2017
	Rescued by Nlgn3 expression in CA1 Pv+ neurons					
R451C KI (Sud^1^)	Sociability: ↓	MWM: ↑	NT	NT		Tabuchi et al., 2007
R451C KI (Sud^1^)	Sociability: ↓	↔	↔	↔		Etherton M. et al., 2011
R451C KI (Sud^1^)	Sociability: ↓	MWM: ↑	NT	NT	Anxiety: ↑	
Backcross w 129S2/SvPasCrl line					Locomotor activity: ↓	Jaramillo et al., 2014
R451C KI (Sud^1^)	NT	NT	↑	Activity: ↑		Rothwell et al., 2014
R451C KI (Sud^1^)	Sociability: ↔	NT	↑	NT	Aggression: ↑	Burrows et al., 2015
R451C KI (Sud^1^)	Increased interest in mating and atypical aggressive behavior by social isolation (Burrows et al., 2017)					
R451C KI (Sud^1^)	Sociability: ↔	NT	NT	NT	Gamma osc: ↓	Cao et al., 2018
	Social novelty: ↓ (Rescued by the optogenetic restoration of gamma osc)					
R451C KI (Sud^1^)	Abnormal visual transitive inference in both lines.					Norris et al., 2019
R451C KI (Sud^1^)	Environmental enrichment reduced anxiety and increased aggression					Burrows et al., 2020
R451C KI (Hei)	Sociability: ↔	↔	↔			Chadman et al., 2008
R451C KI (Hei)	Sociability: ↔	↔	↔	Anxiety: ↔		Jaramillo et al., 2018
				Locomotor activity: ↔		

*FL, fear learning; Motor, motor coordination/learning; MWM, Morris Water Maze; Rep, repetitive behavior; rew/mem, reward/memory; USV, ultrasonic vocalization; OE, overexpression; osc, oscillation; D1, D1R+ medium spiny neuron; D2, D2R+ medium spiny neuron; DA, dopaminergic neuron; PC, Purkinje neurons; Pv+, parvalbumin+ interneuron; ↑, increased; ↓, decreased; ↔, not significant; NT, not tested.*

*Sud^1^: (Tabuchi et al., 2007), Sud^2^: (Tabuchi et al., 2007), Sud^3^: (Rothwell et al., 2014), Hei: (Chadman et al., 2008), Tan: (Tanaka et al., 2010), Bro: (Varoqueaux et al., 2006).*

## Circuit-Specific Functions and Dysfunctions of Nlgn3

**FIGURE 3:**
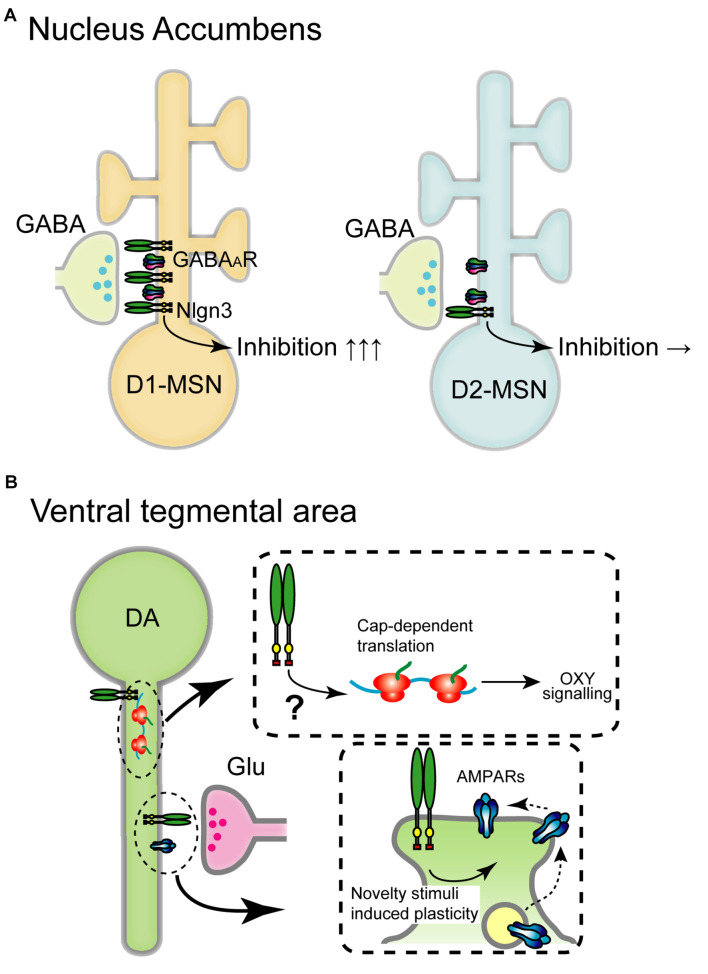
Schematic diagram of the circuit-specific Nlgn3 functions in the mesolimbic pathway. **(A)** Cell type-specific expression of Nlgn3 in the nucleus accumbens. D1 receptor+ medium spiny neurons (D1-MSN, left) in the nucleus accumbens express higher levels of Nlgn3 than D2-MSNs (right) and regulate motor learning. **(B)** Nlgn3 function in the VTA. Nlgn3 expressed in DA neurons regulates cap-dependent translational machinery important for oxytocin (OXY) signaling (upper) (Hornberg et al., 2020) and activity-dependent GluR2-lacking AMPA receptor trafficking (lower) (Bariselli et al., 2018). GABA_*A*_R: GABA_*A*_ receptor.

**FIGURE 4:**
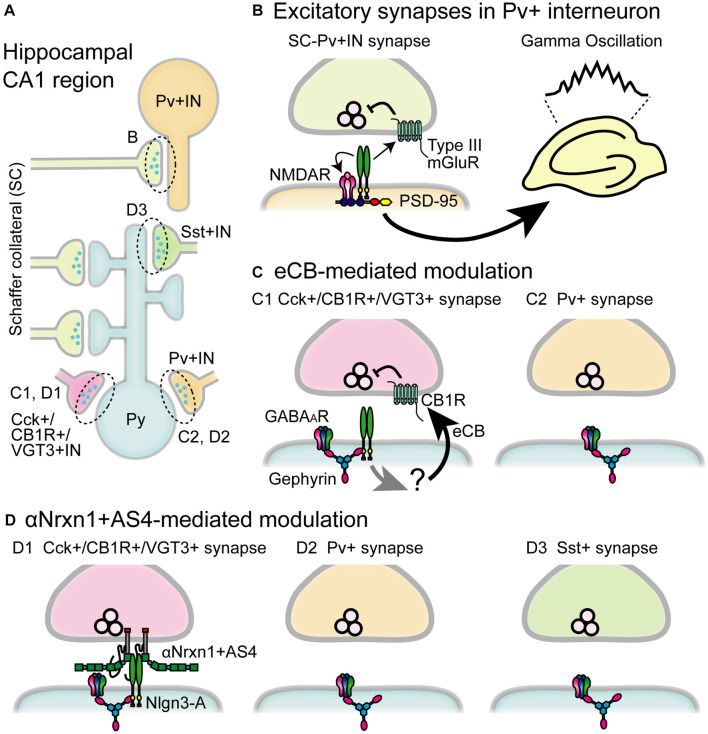
Schematic diagram of the circuit-specific Nlgn3 functions in the hippocampal CA1 region. **(A)** Schematic microcircuits in the hippocampus. Schaffer collaterals (SC) form excitatory synapses on both pyramidal cells (Py) and parvalbumin+ interneurons (Pv+ IN). Distinct classes of interneurons including cholecystokinin+/cannabinoid CB1 receptor+/vesicular glutamate transporter type 3+ interneurons (Cck+/CB1R+/VGT3+ IN), Pv+ interneurons and somatostatin+interneurons (Sst+IN) form inhibitory synapses on pyramidal cells. **(B)** Multiple modes of Nlgn3 regulation in Pv+ interneurons shape hippocampal network activity such as gamma oscillation. Postsynaptic Nlgn3 at SC-Pv+IN synapses regulates NMDA receptor function and retrogradely suppresses release probability through presynaptic group III mGluRs (Polepalli et al., 2017). **(C)** Inhibitory input-specific Nlgn3 regulation. Postsynaptic Nlgn3 regulates Cck+ but not Pv+ synaptic strength via the activation of tonic endocannabinoid (eCB) signaling (Foldy et al., 2013). **(D)** Inhibitory input-specific Nlgn3 regulation through trans-synaptic interaction with αNrxn1+AS4. Nlgn3 is selectively expressed at inhibitory synapses expressing VGT3 and CB1, and regulates inhibitory synaptic transmission with presynaptically expressed αNrxn1+AS4 (Uchigashima et al., 2020a). GABA_*A*_R, GABA_*A*_ receptor.

Recent cell type-specific KD, KO, and rescue approaches, and the development of simultaneous pre- and post-synaptic gene introduction method allows us to deepen our knowledge of synapse-, cell type-, and brain region-specific roles of Nlgn3 on synaptic function and animal behaviors. In this section, we summarize the functional importance of Nlgn3 expressed in different cells and circuits in the brain (Figures 3,4 and Tables 1–3).

### Nlgn3 Function in Striatal Circuit

Aberrant striatal development and connectivity are linked to repetitive behaviors and abnormal reward circuitry in ASD (Hollander et al., 2005; Langen et al., 2009; Di Martino et al., 2011; Langen et al., 2014; Abbott et al., 2018). Striatum is divided into dorsal and ventral regions which include the caudate nucleus and putamen, and nucleus accumbens, respectively. *Nlgn* genes are expressed in both regions, however, *Nlgn3* expression levels vary depending on the cell type and striatal region. *Nlgn3* mRNA levels, but not *Nlgn1* and *Nlgn2*, are significantly higher in D1-MSNs than in D2-MSNs in the nucleus accumbens. In the dorsal striatum, there are no differences in the expression of *Nlgn1-3* (Rothwell et al., 2014). Concomitant to this expression profile, Nlgn3 KO selectively reduces inhibitory synaptic transmission in ventral D1-MSNs but not D2-MSNs without changing excitatory basal synaptic transmission and mGluR-dependent plasticity. Nlgn3-R451C KI mutant mice also display reduced inhibition in D1-MSNs in the nucleus accumbens suggesting that Nlgn3 localizes primarily at inhibitory synapses and regulates D1-MSNs (Figure 3A).

Both Nlgn3 KO and Nlgn3-R451C KI mice display enhanced rotarod performance and stereotypic behaviors (Table 2; Rothwell et al., 2014). Purkinje neuron-specific Nlgn3 KO is sufficient to induce hyperactivity but does not affect motor learning. In contrast, elevated motor learning behavior is regulated by Nlgn3 expressed in D1-MSNs in the nucleus accumbens but not in the dorsal striatum (Rothwell et al., 2014), indicating that ASD-associated behavioral phenotypes are precisely regulated by Nlgn3 expressed in specific brain structures.

### Nlgn3 Function in Dopaminergic Neurons

In VTA DA neurons, cocaine, social reward, and novelty stimuli induce AMPAR-mediated synaptic plasticity by regulating GluR2-lacking AMPAR trafficking (Luscher and Bellone, 2008; Bariselli et al., 2016, 2018). Nlgn3 KO and DA neuron-specific Nlgn3 KD caused the dysregulation of GluA2-lacking AMPAR insertion which correlates with abnormal social behavior (Bariselli et al., 2018; Figure 3B).

Recent studies have highlighted Nlgn3 function in VTA DA neurons on social behavior. Social reward, sociability, and social novelty were reduced by specific Nlgn3 KD in VTA DA neurons (Bariselli et al., 2018). Social novelty deficits in Nlgn3 KO mice were rescued by specific expression of Nlgn3 in VTA DA neurons (Hornberg et al., 2020). Furthermore, Nlgn3 KO dysregulates the translation of oxytocin signaling in VTA DA neurons through the MAP kinase cascade (Figure 3B). This suggests that Nlgn3 is not only a trans-synaptic regulator but also regulates translation and oxytocinergic signaling. It is particularly important to elucidate the localization of Nlgn3 in DA neurons. Where does Nlgn3 localize at excitatory, or inhibitory synapses? Further investigations are required to map the expression of Nlgn3 at distinct synapses.

### Nlgn3 Function in Pv+ Inhibitory Interneurons

Gamma band oscillations implicated in various phases of hippocampal- and cortical-dependent cognitive behaviors (Buzsaki and Wang, 2012; Adaikkan and Tsai, 2020) and dysfunction of gamma oscillations have been found in people with ASD (Rojas and Wilson, 2014). Gamma oscillations are elicited by the firing of fast-spiking inhibitory interneurons and Pv+ inhibitory interneurons, which are the dominant fast-spiking interneuron cell type, to generate gamma oscillations underlying various animal behaviors (Cardin et al., 2009; Buzsaki and Wang, 2012). Importantly, selective deletion of *Nlgn3* gene in Pv+ interneurons and Nlgn3 KO mice display reduced power of gamma oscillations in the hippocampus (Polepalli et al., 2017; Modi et al., 2019; Figures 4A,B). Furthermore, Pv+ interneuron-specific Nlgn3 KO mice exhibit abnormal hippocampus-dependent learning and memory, including fear memory retention and extinction (Polepalli et al., 2017). Dysregulated gamma oscillation was also found in Nlgn3-R451C KI mice (Cao et al., 2018). Fast-spiking interneurons in the medial prefrontal cortex (mPFC) exhibited reduced excitability while optogenetic restoration of Pv+ neuron excitability rescued deficits in social novelty preference. Interestingly, the social submissive behavior observed in Nlgn3 KO mice was rescued by expression of Nlgn3 in Pv+ interneurons, suggesting that Pv+ interneurons may be a primary target causing abnormal phenotypes in Nlgn3 KO mice (Kalbassi et al., 2017). Taken together, these results strongly suggest that Nlgn3 in Pv+ interneurons regulate cognitive behaviors and dysregulation of excitatory and inhibitory balance is one of the endophenotypes of ASD, which offers a new therapeutic approach for social behavior challenges in individuals with ASD.

### Nlgn3-Mediated Retrograde Presynaptic Regulation

Numerous *in vitro* studies that cultured dissociated neurons with non-neuronal Nlgn-overexpressed cells have demonstrated that expression of Nlgns in non-neuronal cells is sufficient to induce presynaptic differentiation (Scheiffele et al., 2000; Graf et al., 2004; Chubykin et al., 2005; Dong et al., 2007; Ko et al., 2009; Etherton M. R. et al., 2011; Sun et al., 2011). These results raise an attractive hypothesis that postsynaptic Nlgns regulate presynaptic functions in a retrograde manner. This hypothesis was confirmed in both Nlgn1 and Nlgn3 (Futai et al., 2007; Wittenmayer et al., 2009; Stan et al., 2010; Shipman et al., 2011). Postsynaptic overexpression of Nlgn1 or Nlgn3 in hippocampal CA1 pyramidal neurons in organotypic slice cultures increased release probability. Our follow-up study suggests that this retrograde modulation is mediated by trans-synaptic interactions between Nlgn and Nrxn (Futai et al., 2013). Overexpression of Nlgn3 can increase the number of presynaptic terminals along the dendrites of hippocampal primary neurons, supporting a role for Nlgn3 protein in synapse formation (Chih et al., 2005). Furthermore, *in vivo* overexpression of Nlgn3+A2 in the neocortex selectively upregulated inhibitory but not excitatory presynaptic proteins such as VIAAT and glutamic acid decarboxylase (GAD) 65 on the overexpressing neurons without increasing postsynaptic gephyrin expression (Fekete et al., 2015). These studies support the retrograde modulation of synaptic function by postsynaptic Nlgn3. However, these studies were restricted to an overexpression approach and no change in presynaptic function has been reported in Nlgn3 KO, Nlgn3-R451C, Nlgn3 R704C, and Nlgn1/2/3 triple KO mice (Varoqueaux et al., 2006; Tabuchi et al., 2007; Etherton M. R. et al., 2011; Chanda et al., 2017).

Importantly, a recent report supports that endogenous Nlgn3 regulates excitatory presynaptic release probability in the hippocampus. Polepalli et al. (2017) found that Pv+ interneuron-specific Nlgn3 KO caused the loss of group III mGluR-mediated inhibition in excitatory presynaptic terminals and increased excitatory synaptic release probability on Pv+ neurons. These results suggest that postsynaptic Nlgn3 negatively regulates presynaptic release probability through presynaptic mGluR signaling (Figures 4A,B). It will be particularly intriguing to elucidate the molecular mechanism that bridges postsynaptic Nlgn3 and presynaptic mGluRs. Interestingly, Nlgn3-mediated retrograde modulation is also found in hippocampal Cck+/CB1+ inhibitory synapses. Cck+/CB1+ inhibitory synapses are persistently suppressed via the constitutive activation of presynaptic CB1R which is mediated by ambient eCB released from postsynaptic neurons (Katona and Freund, 2012). This mode of eCB-mediated suppression at Cck+/CB1+ inhibitory synapses is disrupted in both Nlgn3 KO and R451C KI mice (Foldy et al., 2013), suggesting a critical role of Nlgn3 in tonic eCB-mediated signaling at Cck+/CB1+ inhibitory synapses (Figures 4A,C).

### “Molecular Code” for an Input Cell-Dependent Function of Nlgn3 Protein at Hippocampal Inhibitory Synapses

The most fundamental and intriguing role of Nlgns is the impact of their trans-synaptic interactions on synaptic function. However, elucidating how trans-synaptic interactions shape functional synaptic properties has remained a challenge. To understand the physiological roles of trans-synaptic molecules one must be able to manipulate the expression of these molecules in pre- and post-synaptic neurons simultaneously and then elucidate the functional consequences of this manipulation. We have recently advanced methodology for performing non-overlapping gene transfections which allows us to express Nlgn and Nrxn isoforms in pre- and post-synaptic neurons simultaneously and decipher roles of specific Nlgn3 and Nrxn interactions on hippocampal inhibitory synaptic transmission (Keener et al., 2020b, a; Uchigashima et al., 2020a). Inhibitory synapses are characterized by distinct neurochemical markers specific to input cell types such as VGT3, Pv, and Sst (Pelkey et al., 2017). Nlgn3-A overexpression can selectively potentiate VGT3+ synapses but not Pv+ or Sst+ synapses. In contrast, Nlgn3+ A2 overexpression potentiates neither VGT3+ nor Pv+ synapses, although Sst+ synapses show different phenotypes depending on the context of experiments (timing and duration of overexpression, animal species, and methods to induce action potentials) (Horn and Nicoll, 2018; Uchigashima et al., 2020a).

What mechanism underlies the input cell- and splice isoform-dependent function of Nlgn3-A protein at inhibitory synapses on CA1 pyramidal cells? One promising mechanism is a trans-synaptic interaction of Nlgn3-A protein with a specific Nrxn isoform. Each Nrxn isoform has been hypothesized to regulate synaptic function via trans-synaptic interactions with postsynaptic binding partners in an isoform-dependent manner (Sudhof, 2017a). Interneuron type-dependent transcription patterns of Nrxns have been demonstrated within the hippocampal CA1 region (Fuccillo et al., 2015; Uchigashima et al., 2020a). Notably, αNrxn1+AS4 mRNA is expressed at higher levels in VGT3+ interneurons than in Pv+ or Sst+ interneurons. In hippocampal slice cultures, Nlgn3-A-mediated potentiation at VGT3+ synapses is abolished in VGT3+ interneuron-specific Nrxn1/2/3 triple KO mice. In contrast, this deficit can be rescued by selective expression of αNrxn1+AS4 protein but no other Nrxns including αNrxn1-AS4 and βNrxn3+AS4 protein (Uchigashima et al., 2020a). These results highlight the importance of specific trans-synaptic interactions between Nlgn3-A and αNrxn1+AS4 proteins which underlie Nlgn3-A-mediated potentiation at VGT3+ synapses in an input cell-dependent manner (Figures 4A,D). However, this specific interaction cannot be explained by biochemical studies in which αNrxn1 protein can bind to Nlgn3 protein regardless of the presence or absence of the splice insertion at AS4 (Boucard et al., 2005). βNrxn3+AS4 protein can bind to all four Nlgn3 splice isoforms (Koehnke et al., 2010). Thus, the underlying molecular mechanism to generate trans-synaptic interactions between Nlgn3-A and αNrxn1+AS4 proteins remains elusive. One possible explanation is a co-factor that can interact with Nlgn3-A and αNrxn1+AS4 proteins to regulate their trans-synaptic interaction. Indeed, the trans-synaptic interaction of Nlgn3 and Nrxn proteins can be modulated by PTPδ or MDGA protein (Lee et al., 2013; Pettem et al., 2013; Connor et al., 2016; Elegheert et al., 2017; Gangwar et al., 2017; Yoshida et al., 2021). This modulation might be structurally or functionally affected by the presence or absence of the short amino acid chain encoded by the alternatively spliced sequence. Another possible explanation is a contribution of other alternatively spliced sequences on Nrxn proteins, the function of which has not been well characterized yet. Further investigations are required in the future.

## Functions of Nlgn3 in Non-Neuronal Cells and Outside the CNS

### Nlgn3 in Glial Cells

Although the function of Nlgns has been extensively examined in the context of neurons, recent evidence indicates that Nlgn proteins can regulate glial morphogenesis and differentiation. Astrocytic Nlgn1-3 proteins are required for astrocyte morphogenesis both *in vivo* and *in vitro* (Stogsdill et al., 2017). The role of Nlgn1-3 proteins in astrocyte morphogenesis differs in an isoform- and time-dependent manner (Stogsdill et al., 2017). Additionally, the Nlgn3-R451C mutation affects astrocytic morphology by reducing their branch point number, branch length, and territory in the dentate gyrus (Matta et al., 2020). Furthermore, oligodendrocytic Nlgn3 protein contributes to the differentiation of oligodendrocytes (Proctor et al., 2015) and the density of microglia is increased in the dentate gyrus of Nlgn3-R451C KI mice (Matta et al., 2020). Interestingly, glial progenitor cells from individuals with schizophrenia express significantly lower levels of Nlgn1, Nlgn2, and Nlgn3 mRNA compared with controls (Windrem et al., 2017), suggesting a role for glial Nlgn3 protein in pathophysiological conditions. In Drosophila, Dnlg3, a homolog of vertebrae Nlgn3 protein initially identified as gliotactin, is transiently expressed on peripheral gila and required for the formation of the peripheral blood-nerve barrier (Auld et al., 1995).

### Nlgn3 in Tumors

Nlgn3 protein can function as a cortical neuronal activity-regulated glioma mitogen (Venkatesh et al., 2015, 2017). Nlgn3 extracellular ectodomain is shed from neurons and OPCs likely through ADAM10 in a neuronal activity-dependent manner. The secreted ectodomain of Nlgn3 can act on glioma cells via unknown binding partners and recruits the phosphoinositide 3-kinase (PI3K)-mTOR pathway to promote glioma cell proliferation. Nlgn3 exposure also induces feedforward expression of Nlgn3 protein at the transcriptional and translational levels, resulting in further increases in Nlgn3 protein expression in tumor cells. Importantly, patient-derived pediatric glioblastoma xenografts to the frontal cortex demonstrate a notable inhibition of glioma growth in Nlgn3 KO mice for up to 6 months. Therefore, Nlgn3 mRNA expression levels are inversely correlated with overall survival, providing a strong prediction of survival in human HGG. Interestingly, there is no effect of Nlgn1, Nlgn4X, Nlgn4Y or Nlgn2 on glioma proliferation, and no significant association between Nlgn2 expression and patient survival in adult glioblastoma multiforme (GBM), suggesting a unique function of Nlgn3 in brain tumors.

### Nlgn3 in the Gastrointestinal System

A prevalent concern among people with ASD revolves around gastrointestinal (GI) symptoms such as constipation, diarrhea, and abdominal pain (Holingue et al., 2018). In support of this, non-syndromic ASD models with the deletion or mutation of Nlgn3 gene exhibit abnormal GI function. Nlgn3-R451C KI mice show a faster small intestine transit of carmine red dye administered by oral gavage and increased sensitivity to GABA_*A*_ receptor-mediated modulation (Hosie et al., 2019). Similarly, Nlgn3 KO mice exhibit faster colonic migrating motor complexes (Leembruggen et al., 2020). Morphologically, no differences in the number of many GI neuron subpopulations have been reported in Nlgn3 KO or R451C KI mice, except for an increase in small intestine myenteric neurons in Nlgn3-R451C KI mice (Hosie et al., 2019; Leembruggen et al., 2020; Sharna et al., 2020).

## Future Direction/Conclusion

Given the heterogenous nature and presentation of ASD, identifying therapeutic targets and improving therapeutic effectiveness remains a challenge. Nlgn3 gene is strongly associated with a non-syndromic monogenic form of ASD and for the past decade behavioral analyses with animal models have revealed that Nlgn3 protein at distinct synapses underlie some abnormal phenotypes caused by the disruption of Nlgn3 gene. Knowledge about the molecular mechanisms underlying the heterogeneous expression and function of Nlgn3 protein will help us understand the complex pathophysiological basis of ASD with the potential to create novel therapeutical strategies.

Trans-synaptic signaling between presynaptic and postsynaptic CAMs is a promising mechanism to decipher molecular heterogeneity at synapses. One feasible approach to dissect specific trans-synaptic signaling with synaptic CAMs is based on cellular biological methods to manipulate the expression levels of both presynaptic and postsynaptic CAM variants at targeted synapses (Uchigashima et al., 2020a). This approach revealed specific trans-synaptic signaling between Nlgn3-A and αNrxn1+AS4 isoforms which mediated an input cell-dependent function of Nlgn3 protein at hippocampal inhibitory synapses. However, three fundamental questions remain to be addressed.

First, can trans-synaptic signaling be generalized to other synapses? Although single-cell transcriptomics has demonstrated cell type-specific expression patterns of synaptic CAMs (Fuccillo et al., 2015), it is unclear which alternatively spliced isoforms of synaptic CAM proteins are delivered to and function at a given synapse. Mapping endogenous splice isoforms of Nlgn3 and αNrxn1 proteins at synapses will be critical. Immunostaining with specific antibodies is the most prevalent method to label endogenous proteins. As in the case of the AS5 of Nrxn3, short amino acid sequences at splicing sites can be detected with specific antibodies (Matsuda et al., 2016). However, as the alternatively spliced sequences A1 and A2 of Nlgn3 protein are shared among different Nlgns, they cannot be distinguished from those of Nlgn1 or Nlgn2 protein. The genomic insertion of tag sequences into the gene locus encoding target proteins could also be feasible (Mikuni et al., 2016). Indeed, the N terminus of Nlgn3 protein can be endogenously labeled with green fluorescent protein (Willems et al., 2020). However, the insertion of tag sequences into the A1 and A2 sequences of Nlgn3 protein may affect the binding affinity to Nrxns due to structural changes of the binding interface on Nlgn3 protein. Second, how are splicing events on Nlgn3 gene regulated? Iijima et al. (2011) revealed one important molecular mechanism to regulate an insertion at the AS4 of Nrxns in a neuronal activity-dependent manner. However, no knowledge is available for the molecular mechanism underlying alternative splicing of Nlgn3 or other synaptic CAM genes. Third, what molecular mechanisms allow Nlgn3 to modulate the neuronal homeostatic function beyond a circuit-specific synaptic organizer that mediates trans-synaptic signaling? The discovery of Nlgn3-mediated translational regulation in VTA neurons has opened new avenues to explore novel roles of Nlgn3. It is essential to understand whether this non-canonical function is specific to Nlgn3 and VTA neurons (Hornberg et al., 2020). Taken together, further technical advances should help answer these questions and at the same time allow us to understand the fundamental molecular mechanism underlying circuit-specific normal function and dysfunction of animal behaviors and neurodevelopmental disorders such as ASD.

## Author Contributions

MU, AC, and KF wrote the manuscript. All authors contributed to the article and approved the submitted version.

## Conflict of Interest

The authors declare that the research was conducted in the absence of any commercial or financial relationships that could be construed as a potential conflict of interest.

## Publisher’s Note

All claims expressed in this article are solely those of the authors and do not necessarily represent those of their affiliated organizations, or those of the publisher, the editors and the reviewers. Any product that may be evaluated in this article, or claim that may be made by its manufacturer, is not guaranteed or endorsed by the publisher.
